# An automated platform for simultaneous, longitudinal analysis of engineered neuromuscular tissues for applications in neurotoxin potency testing

**DOI:** 10.1016/j.crtox.2025.100218

**Published:** 2025-01-26

**Authors:** Jacob W. Fleming, Molly C. McCloskey, Kevin Gray, David R. Nash, Vincent Leung, Christos Michas, Shawn M. Luttrell, Christopher Cavanaugh, Julie Mathieu, Shawn Mcquire, Mark Bothwell, David L. Mack, Nicholas A. Geisse, Alec S.T. Smith

**Affiliations:** a Curi Bio Inc., 3000 Western Avenue, Seattle, WA, USA; b Comparative Medicine Department, University of Washington, Seattle, WA, USA; c Department of Neurobiology and Biophysics, University of Washington, Seattle, WA, USA; d Institute for Stem Cell and Regenerative Medicine, University of Washington, Seattle, WA, USA; e Department of Bioengineering, University of Washington, Seattle, WA, USA; f Department of Rehabilitation Medicine, University of Washington, Seattle, WA, USA

**Keywords:** Neuromuscular junction, Engineered skeletal muscle, Engineered tissue models, Potency assay, High-throughput model

## Abstract

Animal models of the neuromuscular junction (NMJ) have been widely studied but exhibit critical differences from human biology limiting utility in drug and disease modelling. Challenges with scarcity, scalability, throughput, and ethical considerations further limit the suitability of animal models for preclinical screening. Engineered models have emerged as alternatives for studying NMJ functionality in response to genetic and/or pharmacological challenge. However, these models have faced challenges associated with their poorly scalable creation, sourcing suitable cells, and the extraction of reliable, quantifiable metrics. We present a turnkey iPSC-based model of the NMJ employing channelrhodopsin-2 expression within the motor neuron (MN) population driving muscle contraction in response to blue light. MNs co-cultured with engineered skeletal muscle tissues produced twitch forces of 34.7 ± 22.7 µN in response to blue light, with a response fidelity > 92 %. Histological analysis revealed characteristic punctate acetylcholine receptor staining co-localized with the presynaptic marker synaptic vesicle protein-2. Dose-response studies using botulinum neurotoxin showed loss of function in a dose- and time-dependent manner (EC_50_ − 0.11 ± 0.015 µg). Variability of the EC_50_ values between 2 different iPSC differentiations of both cell types and 2 users was less than 2 %. Further testing with the acute neurotoxins acetylcholine mustard and d-tubocurarine validated the biological relevance of the postsynaptic machinery of the model. This model marks a meaningful progression of 3D engineered models of the NMJ, providing engineered tissues at a throughput relevant to potency and screening applications with an abundant iPSC cell source and standardized hardware-software ecosystem allowing technology transfer across laboratories.

## Highlights

• Turnkey iPSC-based model of the NMJ with standardized hardware-software ecosystem.
• Dose dependent loss of function with botulinum toxin application for toxin potency.
• *In vivo*-like staining for key pre- and postsynaptic markers.
• Demonstrated wash-in and wash-out sensitivity to fast acting neurotoxins.

## Graphical abstract

**Figure:**
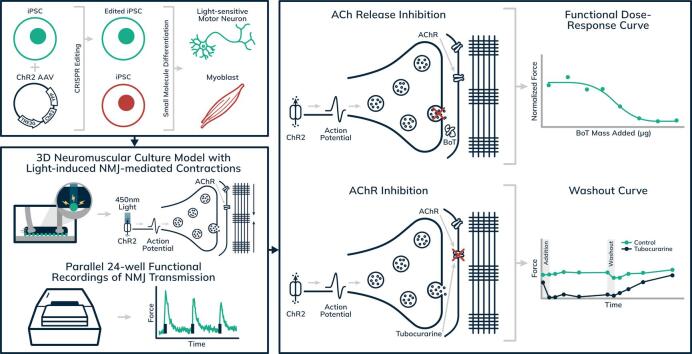

## 1 Introduction

The neuromuscular junction (NMJ) is the essential synaptic connection through which motor neurons control skeletal muscle contraction. In response to incident neuronal action potentials, acetylcholine (ACh) is released from presynaptic vesicles in motor neurons and then binds to postsynaptic nicotinic ACh receptors (nAChR) presented in clusters on the post synaptic muscle membrane. This results in positive ion influx and initiating muscle contraction (reviewed in (Davis et al., 2022). During development, NMJs undergo a process of growth, pruning, and maturation, ultimately forming stable highly specialized structures that include postsynaptic membrane folding and invagination, a robust basement membrane, and several key transmembrane support molecules (Huh and Fuhrer, 2002). Individual muscle fibers are innervated by a single motor neuron, which branches to innervate several muscle fibers creating a single motor unit (Sanes and Lichtman, 1999). Impaired NMJ function is observed in a range of diseases that impact motor neuron health, for example amyotrophic lateral sclerosis (ALS) and spinal muscular atrophy (SMA), or diseases of the muscle fiber such as muscular dystrophy (Lovering et al., 2020, Mercuri et al., 2022, Hardiman et al., 2017).

There also exist toxins that target presynaptic machinery, postsynaptic signaling, vesicle recycling, and neurotransmitter degradation or reuptake, collectively termed neurotoxins. Both synthetic and naturally derived neurotoxins exist, along with a range of industrial/agricultural chemicals with neurotoxic off-target effects. Used broadly in medical practice, as bio/chemical weapons, and encountered accidentally through industrial exposure, this class of molecules are widely studied for a variety of purposes. Neurotoxins that target the presynaptic machinery include botulinum toxin (BoT). BoT cleaves components of the SNARE (soluble N-ethylmaleimide-sensitive component attachment protein receptor) complex involved in presynaptic vesical fusion for release of ACh from the motor neuron. BoT is commonly used for medical and cosmetic purposes. It is a naturally derived biologic product produced by the gram-positive bacterium *Clostridium botulinum*. At least seven BoT serotypes exist (A-G), with each serotype using different presynaptic proteins for its uptake, activation, and target cleavage in the presynaptic membrane (Rossetto et al., 2014, Schiavo et al., 2000, Montal, 2010, Zhang et al., 2017). Other toxins, such as tubocurarine and acetylcholine mustard (AChM), target the postsynaptic machinery. Tubocurarine, a plant-derived neurotoxin, binds competitively to the nAChR at the postsynaptic membrane on skeletal muscle, blocking binding of ACh and thus neuromuscular communication (Bowman, 2006). Its binding is reversible, and it has historically been used in anesthesiology for neurological blockade. AChM is a structural analog of ACh and can bind to both muscarinic and nicotinic AChRs, blocking ACh binding and neuromuscular communication in a partially irreversible fashion due to covalent modification of the active site (Cohen et al., 1991). AChM has a transitory, excitatory effect due to its binding within the ACh binding site, after which receptor desensitization occurs (Hudgins and Stubbins, 1972).

As a large and abundant synapse, animal studies of the NMJ have given a detailed understanding of the effects of disease and toxin action but comparisons to human NMJs have revealed a range of biologically significant differences. Differences such as NMJ morphology (Boehm et al., 2020) and BoT serotype B (BoT/B) potency (Elliott et al., 2019, Strotmeier et al., 2012) highlight the need for high fidelity reproduction of human biology in research models. Progressive diseases, including many neuromuscular diseases, are also challenging to study in animals as longitudinal, quantitative studies can be challenging to obtain and genetic backgrounds often influence disease progression (Hardiman et al., 2017, Van Daele et al., 2023). Additionally, the use of animals in disease modeling and toxin research raises ethical and technical challenges especially associated with throughput and reproducibility (Kabene and Baadel, 2019, Pellett et al., 2019).

To address the challenges associated with animal models of the neuromuscular system, there has recently been a rise in the development and application of advanced *in vitro* modeling. These efforts have led to the establishment of numerous functional cell-based models of skeletal muscles (Capel et al., 2019, Shahriyari et al., 2022, Ebrahimi et al., 2021, Ito et al., 2014, Xu et al., 2020, Huang et al., 2005, Wroblewski et al., 2020, Boonen et al., 2010, Afshar et al., 2020, Tiburcy et al., 2019, Rao et al., 2018, Maffioletti et al., 2018, Shansky et al., 1997). These models have been extended to include the NMJ and are diverse in design, functional readouts, and complexity (Charoensook et al., 2017, Guo et al., 2020, Aydin et al., 2020, Osaki et al., 2018, Rimington et al., 2021, Bakooshli et al., 2019, Yamamoto et al., 2021, Santoso et al., 2021, Vila et al., 2019, Martin et al., 2015, Smith et al., 2013). Many of the models support long-term culture, which is important to ensure sufficient maturation of the NMJ and allow progressive diseases such as ALS to be meaningfully modeled. Models employ functional bioengineered muscle models innervated with motor neurons, which are either cultured in a separate chamber or grown directly with or in close proximity to the muscle constructs. While culturing all cells within a single chamber or device is often technically less challenging, this format is less akin to neuromuscular tissues *in vivo*, where motor neuron axons must project from the spinal cord to the skeletal muscles. This *in vitro* approach also poses challenges to cell-specific activation and recording due to the close proximity of both cell types. Separate chambers allow the utilization of cell-specific media in each compartment, selective stimulation of the desired cell type, and cell-specific recording capabilities. However, these platforms are often difficult to manufacture at scale, suffer from challenges associated with cell seeding and axonal extension, and can be technically challenging to adopt. When considering the replacement of animal models at scale, manufacturability and easy adoption are critical elements to consider when designing and validating novel platforms and often make the difference between adoption and non-adoption of said models.

Early engineered NMJ models primarily relied on spontaneous motor neuron activity (Rimington et al., 2021, Yamamoto et al., 2021) or addition of glutamate (Osaki et al., 2018, Bakooshli et al., 2019, Smith et al., 2013), the natural activator of motor neurons, to specifically excite the motor neuron population. Of the published models only a subset have achieved neuronally directed muscle contraction in response to a temporally controlled stimulus (Osaki et al., 2018, Vila et al., 2019, Santhanam et al., 2018). Spontaneous activity poses challenges in eliciting robust, quantifiable outputs, which are essential for assays that demand a reliable platform readout. Attempted solutions have used electrical stimulation to depolarize biological membranes and trigger action potentials, but these approaches are indiscriminate and will activate both skeletal muscle and motor neurons alike if the cell types are not electrically isolated from one another within a specialized consumable. This requirement brings significant design, manufacturing, reproducibility, and usability challenges. An alternative approach that circumvents these barriers uses optogenetic stimulation to stimulate neuronal action potentials and their concordant activation of muscle contraction (Osaki et al., 2018). This method enables the use of simpler single-chamber models that address the aforementioned manufacturing, reproducibility, and usability advantages of these simpler cultures. Optically excitable motor neurons were demonstrated first by Uzel and colleagues, who transfected the channelrhodopsin-2 (ChR2) gene into mouse embryonic stem cells (ESC) (Uzel et al., 2016). Others followed suit, creating additional stable optogenetic mouse ESC lines (Aydin et al., 2020, Machado et al., 2019, Solomon et al., 2021) and human ESC lines (Steinbeck et al., 2016), or directly transducing neural stem cells (Osaki et al., 2018) or motor neurons (Lin et al., 2019). Recently, some groups overcame the challenges of creating stable optically excitable human induced pluripotent stem cell (iPSC) lines (Vila et al., 2019, Swartz et al., n.d.), which are often intolerant to optogenetics. This technology allows iPSC-derived motor neurons expressing ChR2 to be created at scale through small molecule motor neuron differentiation for use within functional 3D NMJ models.

We have previously established a 24-well plate format model of 3D engineered skeletal muscle tissues (EMTs) that utilizes magnetic sensing for parallel, label-free tissue measurements (Mantarray; Curi Bio Inc., Seattle, WA, USA) (Smith et al., 2022). The platform uniquely enables simultaneous, label-free measurements of tissue contraction force across an entire microplate, thus allowing for repeated recordings from the same tissues over months-long experimental times. This prolonged culture time enables both drug durability and wash-in/-out experiments where effects on muscle function are assessed. In this paper, we extend the existing *in vitro* muscle contractility platform towards neurotoxin and drug screening applications by adding human iPSC-derived neurospheres in the tissue culture. To enable selective and robust stimulation of the neuronal population, we created a stable human iPSC line with doxycycline (Dox)-induced ChR2 expression, which allows for cell-specific blue-light (BL) activation of differentiated motor neurons. Key advantages of this model are in line with the fundamental requirements of preclinical screening platforms and include high reliability, consistently achieving > 95 % success rate in tissue creation, parallelization (all tissues can be simultaneously stimulated and recorded), and design optimization for straightforward manufacturing, automation and adoption by unspecialized operators. The platform demonstrates co-localization of well recognized pre and postsynaptic markers, although this is asymmetric and incomplete indicating immature NMJ formation. In addition, reproducible responses to NMJ antagonists targeting both the pre- and postsynaptic machinery are observed in the model. The results presented here highlight the biological relevance and downstream utility of the platform in preclinical toxicity screening or discovery pipelines.

## 2 Materials and methods

### 2.1 Maintenance of iPSC lines

Urine-derived iPSC lines generated internally, at the University of Washington, were used for the generation of Curi Bio’s commercial myoblasts while WTC-11 iPSCs (Kreitzer et al., 2013, Miyaoka et al., 2014) were used for the generation of TET-ChR2-YFP iPSC line used for motor neuron generation. iPSCs were maintained in mTeSR (Stem Cell Technologies, Vancouver, Canada, 05855) using common precautions for stem cell culture to maintain pluripotency. Detailed supplemental methods include complete protocols.

### 2.2 Generation of TET-ChR2-YFP blue light sensitive line

Working with the University of Washington’s Institute for Stem Cell and Regenerative Medicine Ellison Stem Cell Core, the AAVS1-TRE3-ChR2-YFP construct was generated to produce an insertion at the AAVS1 safe harbor site. The construct was electroporated along with Cas9 protein and guide RNAs to induce insertion. Correct insertion was confirmed by PCR. Detailed supplemental methods include complete protocols.

### 2.3 Differentiation of iPSC-derived motor neurons

Motor neurons were differentiated as described previously (Chun et al., 2024). Briefly, WTC11 iPSCs (Kreitzer et al., 2013, Miyaoka et al., 2014) were passaged onto Matrigel-coated six-well plates and incubated at 37 °C and 5 % CO_2_ in mTeSR until they reached ∼80 % confluency. At this point, cultures were differentiated into regionally unspecified neural progenitor cells using a monolayer differentiation method adapted from Shi et al. (2012) (Shi et al., 2012). These cells were then passaged onto 0.01 % poly-L-ornithine (Sigma-Aldrich, P4957)/5 μg/mL laminin (Sigma-Aldrich, L2020)-coated surfaces and exposed to culture conditions promoting motor neuron differentiation, essentially as described by Amoroso et al. (2013) (Amoroso et al., 2013).

### 2.4 Electrophysiological characterization of motor neuron activity

Whole-cell patch clamp recordings were collected from human iPSC-derived motor neurons at day 35–40 post-induction with both single action potentials and repetitive firing behavior recorded in current-clamp mode.

Population-level function in motor neuron cultures was assessed in 48-well multielectrode array (MEA) plates using the Maestro Pro MEA system (Axion Biosystems, Atlanta, GA, USA). Blue-light stimulation was achieved using the Lumos hardware (Axion Biosystems) in combination with the Maestro Pro system and controlled using the Axis software. Detailed supplemental methods include complete protocols.

### 2.5 Expansion of human dermal fibroblasts

Primary human dermal fibroblasts from Lonza bioscience (Lonza, USA, CC-2511) were thawed, spun (300 *g* for 3 mins) and resuspended in FGM-2 (Lonza, CC-3132). Cells were counted and plated at approx. 3000 cells/cm^2^ into standard cell culture flasks. Medium was changed every 2–3 days and cells were sub-cultured before cells reached 80 % confluence. Cells were banked at p5 in mFreSR (Stem Cell Technologies, 05855) at a density of 3x10^6^ cells/mL for generation of EMTs directly from thaw.

### 2.6 Generation and maintenance engineered skeletal muscle tissues

Engineered muscle tissues (EMTs) were generated within the Mantarray platform (Curi Bio, Seattle, WA, USA, MANTA-24) according to the manufacturer’s protocols (Berry et al., 2023). In brief, iPSC derived skeletal muscle myoblasts and primary human dermal fibroblasts (Lonza, USA CC-2511) were thawed and resuspended independently, spun at 300 *g* for 5 mins, and resuspended to count cells. Cells were remixed at a ratio of 9:1 (myoblasts:fibroblasts) and a density of 420,000 cells per tissue to be cast. The resulting cell pellet was resuspended in: 4 µL 50 mg/mL fibrinogen solution (Sigma-Aldrich, F8360), 24 µL DMEM medium (Gibco, 14190–250), and 12 µL of Matrigel (Corning, 356231) producing a total suspension volume of 40 µL per tissue. The resulting suspension was mixed thoroughly with 20 µL of 100 U/mL thrombin solution (Sigma-Aldrich, T4648) *in situ* within the Mantarray casting consumable to create a molded cellular hydrogel within the casting consumable. Following 80 mins of incubation at 37 °C and 5 % CO_2_ to allow hydrogel formation, 1 mL of SkGM-2 complete medium (Lonza, CC-3245) was added atop the hydrogel within the casting consumable. A further 10 mins of incubation was undertaken to allow hydrogel release from the casting consumable, after which the tissue lattice and attached tissues were lifted from the casting consumable into a media maintenance plate containing 2 mL of SkGM-2 complete medium supplemented with 2 g/L aminocaproic acid (ACA). Following 24 hrs of cell culture, tissues were transferred to 2 mL of Curi Bio iPSC myogenic differentiation medium (Curi Bio, SKM-iPSC-250-D) per tissue, this day was termed day 0. Media was changed every 2–3 days thereafter up until day 10 at which point maintenance of tissues was carried out in Curi Bio iPSC myogenic maintenance medium (Curi Bio, SKM-iPSC-250-M) changed every 2–3 days.

### 2.7 Generation of neurospheres for co-culture motor neuron-skeletal muscle tissues (MN-SkM EMTs)

Neurospheres used for co-culture generation were created using iPSC-derived motor neurons from motor neuron iPSC differentiations at day 18 (Chun et al., 2024). Motor neurons were banked by freezing following differentiation and subsequently used directly from thaw for neurosphere generation. Prior to cell thaw, NMJ casting trenches were treated with anti-adherence solution (Stem Cell Technologies, 07010) according to manufacturer’s direction. Motor neurons were thawed and transferred to conical tubes, basal neuronal media was added dropwise with gentle mixing to prevent osmotic shock in the motor neurons. Cells were counted, spun at 300 *g* for 5 mins, and then resuspended at a density of 2.5x10^6^ cells/mL in complete neuronal maintenance medium plus Y-27632 (Chun et al., 2024). 100 µL of the cell suspension (250,000 cells) was plated into each well of the NMJ casting trench consumable to allow neurosphere generation within the embossed geometry. Following 24 hrs of incubation at 37 °C and 5 % CO_2_, 500 µL neuronal medium was added per well, taking care not to disturb neurospheres within the consumable. Neurospheres were then maintained by partial media changes, removing 500 µL of medium and replacing with 500 µL fresh medium every 2–3 days.

### 2.8 Generation of MN-SkM EMTs

Following 10 days of culture as neurospheres, neurons were used to generate co-culture engineered tissues with 10-day old EMTs. All media was gently removed from the NMJ casting consumable and replaced with a 1 mg/mL type-I rat tail collagen (Sigma-Aldrich, 08–115) plus 5 % Matrigel solution. To generate this collagen solution, acidic rat tail collagen was diluted to 1 mg/mL in DMEM and neutralized dropwise with a 1 M NaOH (Sigma-Aldrich, S2770) solution until a color change to vivid magenta was observed. Matrigel was then added to a final solution volume of 5 %. Following addition of collagen/Matrigel to the NMJ casting trenches, a Mantarray tissue lattice, populated with day 10 EMTs, was introduced into the NMJ casting consumable, submerging the tissues in the hydrogel solution. The co-culture was then incubated for 1 hr, at 37 °C and 5 % CO_2_, and then 1 mL of SkGM-2 complete medium (Lonza, CC-3245) plus neuronal supplements was added atop the hydrogel. The co-culture tissues were maintained *in situ* for 24 hrs and then the medium was replaced *in situ* with iPSC myogenic maintenance medium (Curi Bio, SKM-iPSC-250-M) plus neuronal supplements. A further 24 hrs later, the co-culture tissues were lifted from the NMJ casting consumable and from thereon maintained in 2 mL of iPSC myogenic maintenance medium (Curi Bio, SKM-iPSC-250-M) plus neuronal supplements and 2 µg/mL doxycycline (Sigma-Aldrich, D3447) with changes every 2–3 days.

### 2.9 Calibration and measurement principles of the Mantarray system

In brief, the Mantarray instrument uses an array of anisotropic magnetoresistive (AMR) sensors laid out underneath the 24 well microplate. The AMR sensors detect changes in magnetic field due to the movement of the magnets embedded in the posts attached to the muscle tissues. As the tissues contract, the AMR signals are digitized and a movement force is calculated via simple beam mechanics applied to a known calibration factor (Smith et al., 2022, Berry et al., 2023). The EMT plate is placed into a set position above the magnetometer array via the hardware enclosure, which enables precise and reproducible placement of post head magnets relative to each AMR. Prior to recording, 30 s of calibration data was recorded without the consumable present to account for the baseline the magnetic environment. The tissue plate was then placed within the instrument cradle and the recording commenced. Magnetic field strength at each AMR sensor is recorded and saved. Next, magnetometer data was processed using a magnet localization algorithm that produces post deflection in mm (Curi Bio, PULSE-B0). The resulting distance measurement is used to calculate force which is then reported as a force vs. time curve for each well, which is then used for further analysis.

### 2.10 Measurement of EMTs and MN-SkM EMT contraction

Active twitch force was generated for each tissue as the average force of 30 consecutive contractions evoked through the application of a single biphasic electrical stimulation pulse (90 mA, 5 ms width per phase) at 1 s intervals using a purpose-built electrode assembly (Curi Bio, MA-STM). Kinetic measurements were derived as the average of 30 pulses as for twitch force. Force frequency recordings utilized 2 s pulse trains, separated by 18 s of rest in a single recording. Pulse trains increased in frequency from 1 – 75 Hz (1, 2, 3, 5, 10, 20, 30, 40, 50, 75 Hz) with individual biphasic pulses within trains of 90 mA, 5 ms width per phase. Blue light stimulation was carried out with a 450 nm laser focused onto the center of the tissue (Fig. S1) using monophasic 250 ms pulse trains (90 mA producing 41.7 mW optical power, 8 ms, 100 Hz) spaced at 5 s intervals. Peak force for each tissue was derived from an average of 10 consecutive peaks. Calculation of active peak force, half relaxation time, and time from 10 % contraction to peak was undertaken using Curi Bio’s pulse 3D platform.

### 2.11 Drug treatment of engineered tissues

For all drug treatments, baseline measurements in maintenance medium were undertaken before drug studies began. Tissues were transferred into prewarmed, fresh maintenance medium plus drug treatments as specified in Results – 3.5, 3.6. Washout was achieved by transferring treated tissues into fresh, prewarmed maintenance medium without drug treatment. BoT/A (Metabiologics, WI, USA, #100) and BoT/B (Metabiologics, #200) complex was purchased as a suspension in PBS at 1 µg/µL. Acetylcholine mustard (Sigma-Aldrich, C001) and Tubocurarine were purchased in powder from (Sigma-Aldrich, T2379), and resuspended in sterile, Ultrapure H_2_O at stock concentrations of 10 mM and 50 mM, respectively. Stocks were diluted in PBS to 100X concentrations prior to final dilution into medium.

### 2.12 Phase contrast microscopy for macro morphological appearance of engineered tissues

Phase contrast images of live tissues were collected through an automated job within Nikon NiS elements software that enables collection of images of all 24 tissues within a culture plate. A 2304 x 800 px frame from a 2X magnification image was collected from a Nikon Eclipse Ti2 microscope for each tissue.

### 2.13 Immunohistochemistry and confocal fluorescence microscopy

Tissues were fixed overnight in 2 mL of 4 % PFA (Santa Cruz Biotechnology, sc-281692) and transferred subsequently to PBS for storage at 4 °C. Tissues were removed from the tissue lattice and blocked/permeabilized in 1 % BSA (Millipore, 82–100-6) with 0.2 % Triton-X 100 (Sigma-Aldrich, T8787) in PBS for 1 hr on a rocker. Following blocking/permeabilization tissues were exposed to primary antibodies (see table S2) overnight at 4 °C in blocking buffer, on a rocker. Antibody solutions were then removed, tissues washed 3 times in PBS and secondary antibody solutions (see table S2) applied for 2 hrs at RT in PBS, on a rocker. Tissues were then washed 3 times in PBS and mounted for imaging. Imaging was undertaken on a Nikon A1R with Yokogawa W1 spinning disk head.

### 2.14 Statistical analysis

For all figures, methods of data display are indicated in the figure legend alongside group replicate numbers. In text within 3, 4 data are presented as mean ± standard deviation. Statistical analyses were carried out in Prism 10. One-way analysis of variance (ANOVA), repeated measures ANOVA, or ANOVA on ranks were used depending on data normality and experimental design. Sphericity was not assumed and corrected (Geisser-Greenhouse) where appropriate. *Post-hoc* tests, two-tailed *t*-tests or Mann-Whitney-U tests depending on data normality, with Sidak/Sidak-Holm correction for multiple comparisons was used to compare data sets. For single group comparisons, significant differences between groups were evaluated using either unpaired two-tailed *t*-tests or Mann-Whitney-U tests depending on data normality. EC_50_ values were produced through non-linear least squares fitting of a 4-PL dose curve function to dose response data. The standard deviation of the fit parameter is presented as the error of this value. For all dose responses the data is expressed normalized between 1 and 0. With 1 representing the mean response at the lowest drug dose and 0 the mean response at the highest drug dose. In all experiments, a p value of less than 0.05 was considered statistically significant.

## 3 Results

### 3.1 Co-culture MN-SkM EMT formation using *in situ* neurosphere creation

**Fig. 1:**
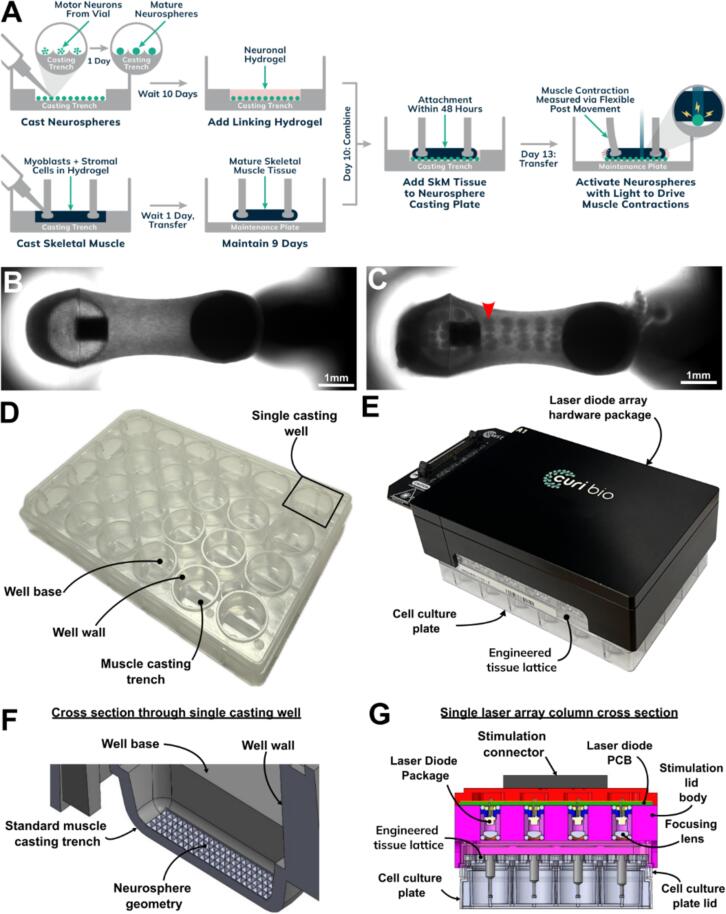
**Systems, protocols and consumables used for the creation and stimulation of MN-SkM EMTs (A)** Schematic illustration of MN-SkM EMT creation. **(B-C)** 2x phase micrographs showing skeletal muscle only EMT and MN-SkM EMT respectively. Scale bars represent 1 mm. Red arrowhead in panel C highlights an adhered neurosphere, these appear in a repeating pattern across the MN-SkM EMT. **(D)** Photograph of 24-well casting plate showing individual casting wells with casting trenches within. **(E)** Photograph of laser diode array hardware package placed atop a cell culture plate and engineered tissue lattice. **(F)** CAD cutaway photograph illustrating neurosphere creation geometry embossed upon neurosphere casting area within a casting trench. Photograph shows cross section through a single well of a 24-well consumable, each well displaying identical characteristics. **(G)** Laser diode based 24-well stimulation system. Figure shows cross section through single column (1 of 6) of device mounted on a 3D tissue cell culture consumable.

To enable high fidelity, high throughput creation of co-culture tissues, we developed a casting process that allows creation and co-culture of > 100 neurospheres with mature EMTs without the need for direct neurosphere handling. Neurospheres were formed through aggregation under gravity in microwells (Fig. 1D) within a casting trench and allowed to mature for 10 days in parallel to EMT formation (Smith et al., 2022). On day 10, neuronal media was removed and replaced with a collagen-matrigel hydrogel into which EMTs affixed to the tissue lattice were submerged (Fig. 1A). This process allows for the creation of 24 co-culture tissues in under 20 min with a casting success rate greater than 95 %, thereby ensuring reliable and reproducible engineered tissue creation. Additionally, we observed that co-culture creation in this manner controlled the placement of neurospheres with high positional fidelity, as the neurospheres in the co-culture were repeatably seen to adopt a grid-like pattern mirroring the neurosphere microwells within the casting consumable (Fig. 1C). To produce specific activation of neurons only, we adopted an optogenetic approach allowing for cell type specific activation dependent upon the expression of ChR2. To enable high-throughput stimulation and recording of co-culture tissues by blue light, an assembly of laser diodes within a 24-well array was designed and manufactured (Fig. 1E). This assembly was designed such that the blue light spot (2.23 x 0.93 ± 0.12 x 0.02 mm, Fig. S1) produced by the laser diode was centered on the EMT tissue (±0.25 mm) within the well below the diode. This provided a nominal maximal stimulation power of 25.6 mW/mm^2^. Together the microwell casting plate (Fig. 1D) and laser diode stimulation assembly (Fig. 1E) allowed reliable creation with independent parallel stimulation of co-culture MN-SkM EMT.

### 3.2 iPSCs containing inducible ChR2 transgenes can be differentiated into functional and blue light activatable motor neurons

**Fig. 2:**
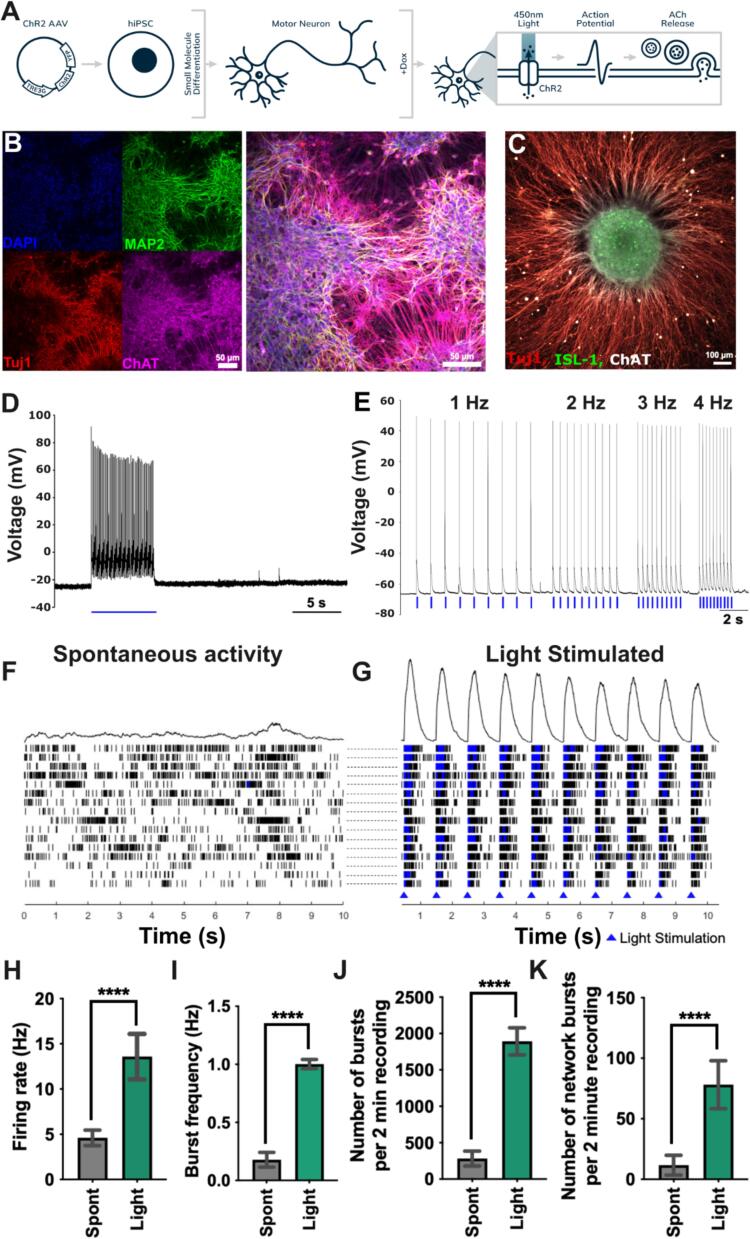
**iPSCs containing inducible ChR2transgenes can be differentiated into functional, blue light activatable motor neurons (a)** Human iPSCs were transduced with a viral construct containing doxycycline-inducible channelrhodopsin 2 (ChR2). iPSCs were differentiated into motor neurons and treated with doxycycline to enable specific activation of the motor neuron population in the NMJ system using blue light. **(B)** Immunostaining of motor neurons in 2D. Scale bars represent 50 µm **(C)** Immunostaining of motor neurons in neurospheres following 24 hrs on adherent surface**.** Scale bars represent 100 µm respectively. **(D)** Example whole-cell patch clamp trace of motor neuron action potential firing train in response to prolonged blue light stimulation. **(E)** Representative whole-cell patch clamp trace of motor neuron action potentials in response to blue light stimulation pulses increasing from 1 Hz to 4 Hz. **(F)** Representative spontaneous activity across multielectrode array (MEA) recordings from a single well containing 16 individual electrodes. **(G)** Representative blue light stimulated activity across multielectrode array (MEA) recordings from a single well containing 16 individual electrodes. Blue pyramid marks indicate incident blue light. **(F-G)** The top of the panel shows a histogram of activity across all electrodes over time, and below a raster plot, with each line a readout from a single electrode. Blue on the raster plot indicates burst firing. **(H)** Firing rate, **(I)** burst frequency, **(J)** number of bursts per 2 min recording, **(K)** and number of network bursts per 2 min recording for MEA measurements. **(H-K)** Mean values ± S.D. are shown for spontaneous activity (Spont – Gray) and blue light stimulation (Light – Green). For all time points n = 48 wells. **Abbreviations**; **DAPI** – 4′,6-diamidino-2-phenylindole nuclear marker, **MAP2** – Microtubule associated protein 2, **Tuj1** – Beta III tubulin, **ChAT** – Choline acetyltransferase, **ISL-1** – islet-1.

To enable selective and robust blue light activation of the neuronal population, we created an iPSC line with stable, doxycycline-induced ChR2 expression and differentiated these cells into motor neurons using previously established protocols (Guo et al., 2020, Chun et al., 2024, Smith et al., 2021) (Fig. 2A, Fig. S2). We validated proper insertion of the viral construct into the iPSCs via PCR (Fig. S2 C-F). To evaluate any potential impacts of ChR2 insertion on the motor neuron differentiation, we employed immunostaining to check for neuronal and motor neuron markers (Fig. 2B). The gene-edited iPSC-derived motor neuron populations demonstrated dendritic expression of microtubule-associated protein 2 (MAP2), axonal expression of β-III-tubulin (Tuj1), and were positive for the cholinergic marker choline acetyltransferase (ChAT), as expected. Additionally, we validated neurosphere cultures via immunostaining for the motor neuron specific nuclear marker islet-1 (ISL-1), expressed in neurosphere core; Tuj1, expressed in the axonal projections; and ChAT at the axon terminals (Fig. 2C). Next, we utilized electrophysiology techniques to confirm gene edited iPSC-derived motor neurons were responsive to blue light stimulation after addition of 2 µg/mL doxycycline and that insertion of ChR2 had no impact on electrophysiological properties. Representative traces of whole cell patch-clamp data for electrically-stimulated edited and unedited motor neurons demonstrated similar action potential waveforms in response to electrical stimulation (Fig. S2 G), and similar action potential properties demonstrating mature cell membranes (Fig. S2 I-J). Further, representative single cell recordings of motor neurons demonstrated repetitive firing in response to prolonged blue light activation (Fig. 2D, Fig. S2 H), and clear generation of action potentials following incident blue light, even up to stimulation frequency of 4 Hz (Fig. 2E). Multielectrode arrays (MEA) were used to compare spontaneous motor neuron activity to BL-activation using motor neuron population measurements. Representative traces of motor neuron activity across electrodes in a single well demonstrate neuronal firing was well synced in response to light stimulation (Fig. 2F). Quantification of these recordings show BL-activation improved overall firing rate (4.60 ± 0.86 Hz vs 13.59 ± 2.51 Hz, p < 0.0001). We also evaluated maturation of neuronal networks by measuring burst activity, which is defined as a recording period where the time between detected depolarizing spikes is under 100 ms for at least ten consecutive spikes (Kapucu et al., 2012, Chiappalone et al., 2005). MEA data indicated BL-stimulation improves all metrics, including burst frequency (0.18 ± 0.06 Hz vs 1.00 ± 0.04 Hz, p < 0.0001), number of bursts (282 ± 103 vs 1892 ± 186, p < 0.0001), and number of network bursts (12 ± 8 vs 78 ± 20, p < 0.0001) compared to spontaneous activity (Fig. 2G). Thus, iPSCs containing inducible ChR2 transgenes can be differentiated into functional motor neurons that are BL-sensitive, enabling robust neuronal activation that can be used within the presented NMJ system.

### 3.3 MN-SkM EMT demonstrate neuronally evoked force generation

**Fig. 3:**
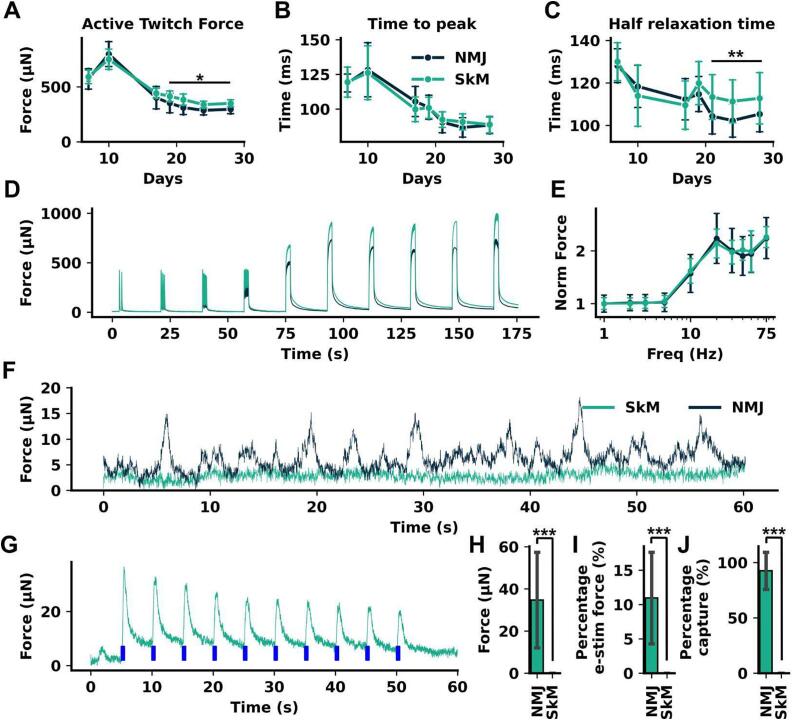
**MN-SkM EMTs show functional responses to electrical stimulation and neural drive (A-C)** Active twitch force, half relaxation time, and time from 10 % contraction to peak respectively across time. Mean values ± S.D. are shown for MN-SkM EMTs (NMJ – Blue) and skeletal only controls (SkM – Green). For all time points NMJ group n = 59 and SkM group n = 22 across 2 technical repeats. **(D)** Average force frequency trace for MN-SkM EMT (green) and SkM EMT (blue) tissues illustrating increased force generation at higher frequencies. **(E)** Average peak height for each frequency normalized to 1 Hz. Graph displays mean ± S.D. **(F)** Representative spontaneous activity from MN-SkM EMT and SkM EMT tissues across a 1 min period. **(G)** Representative blue light driven functional response from MN-SkM EMT. Trace displays force vs time with blue bars (to scale in time) indicating blue light pulses. **(H-J)** Peak force, percentage of electrically evoked force, and percentage of functional responses observed within 250 ms of blue light pulse (percentage capture) respectively. Graphs display mean tissue values ± S.D. For NMJ group n = 59 and SkM group n = 22 across 2 technical repeats.

To examine the effect of neuronal co-culture on EMT function, SkM-only EMTs were compared to MN-SkM EMTs in the same media conditions. At Day 10, prior to neuronal addition, EMTs in SkM-only and MN-SkM EMT groups (pre-neuron addition) showed no significant difference in active twitch force (751 ± 92 µN vs 799 ± 113 µN, p = 0.92, Fig. 3A), time to peak force from 10 % contraction (0.118 ± 0.010 s vs 0.114 ± 0.014 s, p = 0.97, Fig. 3B), or half relaxation time (0.128 ± 0.020 s vs 0.126 ± 0.019 s, p > 0.99, Fig. 3C), showing stable EMT populations before co-culture creation. Following co-culture creation, significant differences in twitch force from day 19 onwards were observed (p < 0.03, 9 days post neuron addition), with an average reduction in active twitch force for MN-SkM EMT tissues of 16 % between day 19 and day 28. Significant reductions in half relaxation times were also observed from day 21 (p < 0.007), with an average reduction of 7.5 % from day 21 to 28. No significant changes in times to peak were observed at any time point. In addition to characterization of individual twitch curves, we examined the force frequency relationship of MN-SkM EMT and SkM EMTs at day 28. As innervation is known to influence fiber type and tissue maturity, both twitch/tetanus ratios and summation transition frequency may be affected by innervation. All tissues produced positive force frequency relationships with stimulation trains of 2 s length and frequencies between 1 and 75 Hz. We observed no difference in twitch/tetanus ratios for SkM vs MN-SkM EMTs at maximum stimulation frequency (2.24 ± 0.38 vs 2.25 ± 0.20) or the apparent transition frequency of these tissues (Fig. 3D, E). SkM-only EMTs show stable baselines with no clear spontaneous activity present. However, a subset of MN-SkM EMTs displayed clear spontaneous activity without stimulation within the prior 24 hrs (Fig. 3F). Spontaneous activity did not appear consistently in all MN-SkM EMTs, but rather a subset of MN-SkM EMTs displayed this behavior. As motor neurons used within the MN-SkM EMTs express ChR2, MN-SkM EMTs would be expected to contract following exposure to blue light. Fig. 3G shows a representative force trace for an MN-SkM EMT undergoing blue light stimulation showing clear contractile responses following incident blue light. Quantification of these responses showed an average twitch force of 34.7 ± 22.7 µN for MN-SkM EMTs with no detectable response from SkM tissues. Blue light evoked force as a percentage of electrically evoked twitch force from the same tissue produced values for MN-SkM EMTs of 11.0 ± 6.6 %. Finally, capture rate, defined as a contractile response within 0.5 s of blue light onset (0.25 s after end of blue light pulse train), for MN-SkM EMTs was 92.5 ± 16.7 %.

### 3.4 Histological examination of MN-SkM EMTs shows co-localization of key pre- and postsynaptic markers

**Fig. 4:**
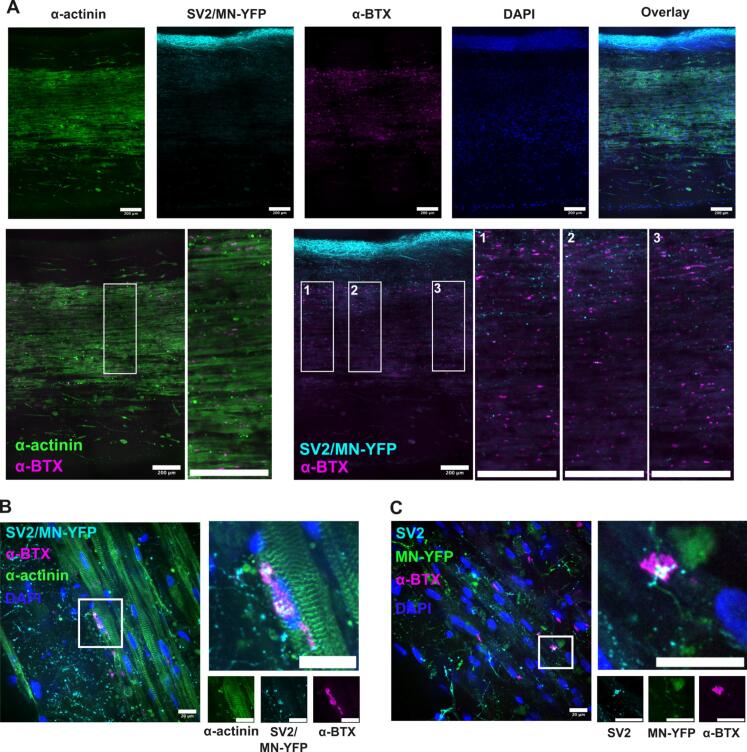
**MN-SkM EMTs present histological evidence of NMJ formation: (A)** Maximum projection images from tiled 20x z-stack micrographs capturing the full width of a single MN-SkM EMT. Single channel false color images are shown first, followed by all channel overlay. Second row shows dual channel overlays (channels as indicated) with zoomed images for box-bounded regions. All images display 200 µm scale bars. **(B-C)** Maximum projection images from 60x z-stack micrographs showing co-localization of key NMJ markers. 4 channel overlay (left), zoomed image from within the highlighted region (right) with single channels (below). All scale bars 20 µm. **Abbreviations**; **MN-YFP** – Motor neurons expressing yellow fluorescent protein, **SV2** – Synaptic vesicle protein 2, **α-BTX** – Alpha bungarotoxin, **SV2/MN-YFP** – Channel co-stained with both SV2 and MN-YFP.

The *in vivo* NMJ has a well understood 3D structure with well-defined pre- and postsynaptic markers. Here we examined SV-2, the presynaptic protein involved in vesicle fusion with the neuronal presynaptic membrane, and the AChR as a post synaptic marker using α-bungarotoxin (α-BTX) as a labelling agent. Additionally, all neurons were labelled with a genetically encoded yellow fluorescent protein (YFP) to visualize neuronal cell bodies and axons. Finally, α-actinin, the z-disk protein, was used to examine myotube cytoskeletal assembly and visualize myotubes within the MN-SkM EMTs. Confocal tile scans of complete tissue sections (Fig. 4A) show a muscle-dominated central region with a peripheral population of neuronal cells densely packed on the outer edge of the tissue (Fig. 4A). This is consistent with the casting process in which an SkM-only EMT is encased by a neuronal cell containing hydrogel. AChR clusters were observed along the length of the muscle portion of the tissue with clear punctate cluster staining on myotubes with even distribution throughout the images taken (Fig. 4A, α-actinin and α-BTX inset). To observe instances of NMJ formation, overlays of SV-2 and α-BTX have been used. Co-localization appears throughout the images, although only a small percentage of α-BTX clusters appear to have an associated SV-2 positive structure overlayed, suggesting relatively low levels of innervation of available AChR clusters (Fig. 4A, SV2/MN-YFP and α-BTX insets). High magnification imaging of AChR clusters that display SV2 co-localization reveals punctate α-BTX staining on striated myotube membranes (Fig. 4B) with SV2 co-localization only partially associated with underlying AChR plaques (Fig. 4B and C). This staining demonstrates clear pre- and postsynaptic membrane marker co-localization but is suggestive of immature NMJs where full sealing of the synaptic cleft and complete pre- and postsynaptic membrane symmetry is yet to be achieved.

### 3.5 Application of botulinum neurotoxin complex A reduces neuronal evoked function in a time and dose dependent manner

**Fig. 5:**
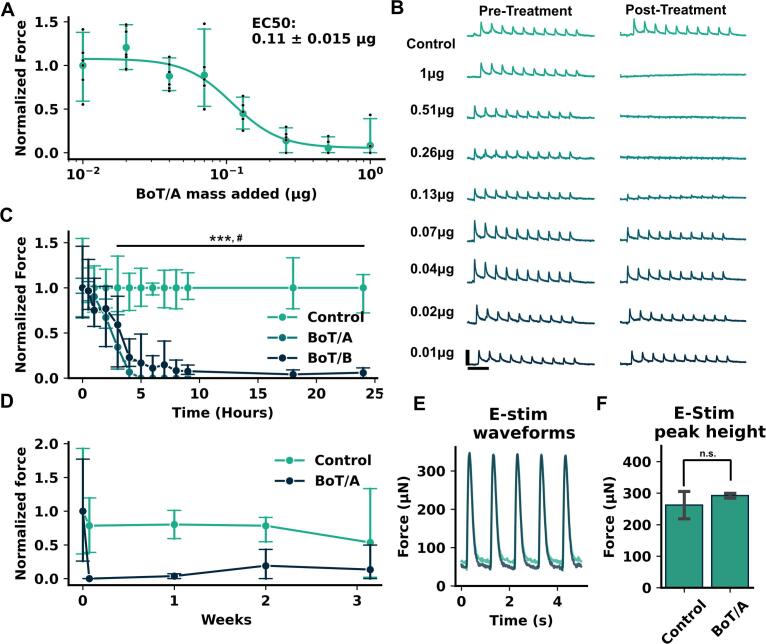
**MN-SkM EMTs show dose- and time-dependent functional blockade in response to botulinum toxin (BoT). (A)** 8-point dose response curve for force vs BoT/A dose following 24 hrs of BoT/A exposure. Values normalized to 10^-2^ µg mean peak force value. Graph displays mean values ± 95 percentile interval (green points) and individual tissue values (grey points), plus a fitted 4PL curve (green line). EC50 value displayed ± S.D. of estimate. Each dose point contains at least n = 6 tissues from 2 technical repeats. **(B)** Representative force traces in response to 10 blue light stimulations at 5 s intervals for control tissues and each dose value within dose response curve pre and post BoT exposure. Scale bar in Y represents 50 µN, scale bar in X represents 1 s. **(C)** MN-SkM EMT response to blue light vs time following BoT/A and BoT/B exposure of 10 µg. Graph displays force normalized to control mean at given timepoint, points represent mean values ± 95 percentile interval. For each condition minimum n = 3 tissues. **(D)** Long term recovery of force following BoT exposure of 1 µg. Graph displays force normalized to control mean at given timepoint, points represent mean values ± 95 percentile interval. For each condition n = 3 tissues. **(E)** Representative waveforms from tissues in response to electrical stimulation following 24 hrs 1 µg BoT exposure. Green – Control, Blue – BoT. **(F)** Quantification of electrical stimulation response following BoT exposure. Bars represent mean ± S.D.

To demonstrate that EMT contraction in response to blue light was driven by the release and recognition of ACh, rather than a non-specific interaction between the co-cultured motor neurons and skeletal muscle, we treated MN-SkM EMTs with botulinum toxin complexes. Treatment with BoT/A for 24 hrs showed a clear dose dependent reduction in blue light evoked force (Fig. 5A). Toxin amounts above 0.26 µg per tissue effectively eliminated blue light responses (Fig. 5B), with an EC_50_ value for the toxin complex of 0.11 ± 0.015 µg per tissue. Fig. 5A displays aggregated data from 2 experiments carried out by separate users and using distinct neuronal and skeletal muscle differentiations as cell sources. Fig. S4 shows individual EC_50_ values for each experiment (0.113 µg and 0.111 µg), showing reproducibility of this response across stem cell differentiations and users. As BoT complexes act by multistep uptake and activation mechanisms, a relatively slow appearance of neuromuscular blockade is expected. To examine this, we applied 10 µg of BoT/A or BoT/B to tissues and examined blue light responses across time, with responses normalized to control tissues. Both toxin complexes significantly reduced function within 3 hrs, with BoT/A reducing function to 34 % and BoT/B 59 % of control tissues (p = 0.003, p = 0.013). Continued action of BoT/A completely ablated blue light evoked function by 5 hrs post treatment while BoT/B reduced function to < 4 % of controls by 24 hrs. As BoTs act only at the synaptic terminal and not in a manner that reduces neuronal viability, we expected to see some level of reinnervation following removal of the toxin. Indeed, after complete loss of function, a subset of BoT/A showed blue light evoked function following 2 weeks of maintenance in the absence of toxin, while control tissues remained relatively stable throughout this period (Fig. 5D). Toxin recovery for BoT/B treated tissues was not assessed. To ensure that BoT/A treatment did not disrupt underlying muscle function we performed electrical stimulation of treated and untreated MN-SkM EMTs following 24 hrs of intoxication. Waveforms for the two groups showed no change (Fig. 5E), and quantification of active twitch force in response to electrical stimulation showed no significant difference between in and xicated and control tissues (p = 0.22).

### 3.6 Fast acting post synaptic toxins show transient blockade of neuronally evoked function in washout drug studies

**Fig. 6:**
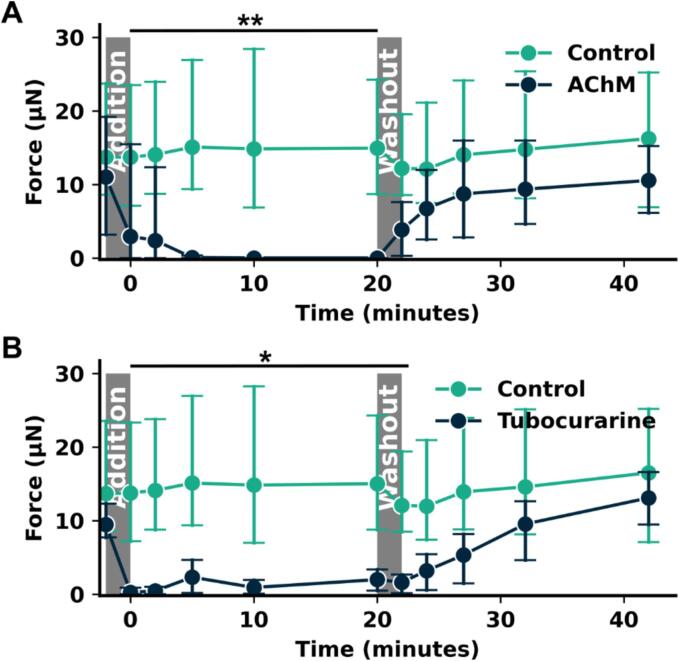
**MN-SkM EMTs show reversible sensitivity to fast acting small molecule antagonists of the NMJ. (A-B)** Blue light evoked force across time for NMJ tissues treated with **(A)** Acetylcholine mustard at 10 µM (AChM) or untreated (Control) or **(B)** d-Tubocurarine at 0.1 µM. Addition and washout of drugs was achieved through complete media change taking approximately 2 mins, indicated on graphs. Graphs display mean ± 95 % percentile interval. For all points n ≥ 4 individual tissues.

To further validate the biological validity of the model and provide evidence of its utility in studies with fast acting neurotoxins, we examined the effect of two postsynaptic toxins that target the AChR, AChM and Tubocurarine. Toxins were added to a subset of wells in a second 24-well plate, into which all 24 MN-SkM EMTs were transferred during the addition stage; for washout, the same process was undertaken to move MN-SkM EMTs from drug treatment to drug-free medium. Two mins were allowed for this process of addition and washout and is included in the time axis for Fig. 6. Controls remained stable throughout the 44 mins experiment across both addition and washout medium changes, with a mean value of 14.1 µN across the experiment. For AChM (Fig. 6A) and Tubocurarine (Fig. 6B), blue light-evoked force diminished immediately following drug addition (p = 0.0037, p = 0.0005) and remained depressed (<10 % AChM, <12 % Tubocurarine) throughout the treatment period (p < 0.0037, p < 0.0013). Following washout, AChM treated tissues remained depressed by an average of 20 % across the final 4 timepoints, although these differences were statistically insignificant compared to control (p = 0.064). Tubocurarine-treated tissues exhibited statistically significant reductions in function immediately post washout (p = 0.016), with function trending linearly towards control levels across the 20 mins washout period. Tubocurarine-treated tissues returned to 82 % of control tissues by the end of the washout period.

## 4 Discussion

Engineered human tissue models hold the potential to transform biological research, by enabling mechanistic studies in preclinical pipelines, allowing scalable potency testing for complex therapeutics and facilitating safety testing of known and novel compounds. However, realizing such potential requires these models to be scalable, turnkey, and reproducible across different users and laboratories. Here we present a model that addresses these challenges and demonstrates key biological hallmarks of functional NMJs, such as neuronally directed muscle contraction, histological colocalization of pre- and postsynaptic markers, and functional blockade using well-characterized NMJ toxins.

The difficulty of co-culturing mature neurons with skeletal muscle within functional systems has been a major barrier to the high-throughput fabrication and use of iPSC-derived MN-SkM EMT models. Previous work has shown the benefits of neurospheres, as the aggregation of MNs into spheroids increases expression of key MN markers and provides extremely robust neuronal extension in adherent culture matrices (Osaki et al., 2018, Rimington et al., 2021, Bakooshli et al., 2019). However, the technical challenges and labor-intensive processes of directly handling neurospheres stand in the way of neurosphere utilization for high-throughput applications. We addressed this challenge by developing an *in situ* spheroid generation approach (Fig. 1A), eliminating the need to manually transfer the spheroids into a co-culture system. This approach simplifies the process to a one-step, time-efficient casting protocol, consistently yielding high co-culture success rates > 95 %, even with users lacking training or experience in either 3D tissue culture or bioengineering. Compared to models requiring direct spheroid handling, our approach significantly improves usability and provides a level of spatial control of neurospheres during co-culture creation, which, although not explicitly tested, likely improves reproducibility over random neurosphere placement. Although conceptually in line with previously published work (Rimington et al., 2021, Bakooshli et al., 2019), the enhanced usability of this model realistically enables the generation of large numbers of tissues required for meaningful applications within preclinical and clinical testing pipelines. To further support the high-throughput potential of our approach, we engineered a laser diode array uniquely designed to apply precisely targeted blue light to tissues within the tissue lattice at high intensities (>20 mW/mm^2^). This array allows simultaneous stimulation of all tissues, extending parallelization beyond the typical well-by-well measurement approaches and reducing data-acquisition timeframes by a factor of 24, a necessary improvement for handling hundreds of tissues in a scalable fashion. This approach reduces the burden on the operator by lowering the prior experience required and improving time requirements for recording, however it does require specialist consumables and hardware. This tradeoff is likely best tolerated by users with a priority on large tissue number and constrained time resource, while high skill users with the ability to prototype consumable items in house may see less benefit from the presented approach.

The presented model employs a direct, bulk co-culture approach in which specific activation of the MNs through broad field electrical stimulation is not possible. Therefore, we developed an iPSC cell line with inducible expression of the blue light sensitive channel ChR2 to allow specific activation of the neuronal population even in direct co-cultures with SkM. We confirmed that the genetic modification did not disrupt the differentiation process of these cells, through a small molecule-directed differentiation process by identifying the expression of the MN markers ISL-1 and ChAT (Fig. 2B,C) in the differentiated population. We also confirmed that these cells formed neurospheres with robust axonal extension (Fig. 2C), key for the spheroid-based approach described in section 3.1. Patch clamp and MEA field potential recordings both confirmed that our iPSC-derived, ChR2-gene-containing neurons produced action potentials in response to incident blue light (Fig. 2D-G) when induced to express ChR2. Many of these action potentials showed classical high-frequency burst train profiles (Fig. 2D), although some patch clamp recordings showed drooping burst train profiles suggesting a subset of neurons within the differentiated populations have not reached full electrophysiological maturity (Fig. S2H). Expression of ChR2 allows for the simplification of co-culture creation, a key aspect of the presented model, but also represents the most significant limitation of the study. Only cells expressing a light sensitive opsin are compatible with the presented system, limiting its utility to genetically edited lines. Although this constraint poses minimal challenges for neurotoxin testing, where healthy control neurons are sufficient, it becomes more significant for modeling genetic diseases with diverse genetic backgrounds, such as ALS (Hardiman et al., 2017, Van Daele et al., 2023). These studies would require generating large numbers of iPSC lines carrying both disease-associated mutations and the inducible ChR2, which may be impractical. To address this, future work will explore viral transduction approaches to deliver opsins to neuronal cell types (Osaki et al., 2018, Duong et al., 2019), broadening the utility of the system and allowing applications within disease modeling using representative populations to be undertaken. Additionally, the use of iPSC derived MNs allows the generation of a stably edited cell line, however users without iPSC differentiation experience and ready access to primary cells would also benefit from viral transduction approaches to remove the reliance of the model on iPSC derived cells.

Bulk co-culture without compartmentalization of cell types requires a co-culture medium which supports both SkM contractile function and neuronal function. Development of media that is sufficient to maintain two cell types simultaneously is often a significant challenge when developing new *in vitro* models, especially those derived from iPSCs. Here, we successfully solved this challenge by using a commercially available SkM muscle media known to support excellent 3D SkM muscle tissue function with the addition of neuronal factors to support neuronal function. The medium is shown to maintain muscle function across time (Fig. 3A-C) and allow innervation (Fig. 3F-J) showing a successful approach to medium composition. Previous work has shown that co-culture of SkM with MN facilitates additional muscle development with 3D tissues producing higher forces or improved contractile kinetics or both (Yamamoto et al., 2021, Martin et al., 2015, Larkin et al., 2006). We did not observe broad changes to functional muscle phenotypes, and so did not examine SkM morphology, identifying only improvements in ½ relaxation times (Fig. 3C). Average twitch force, time to peak and force frequency relationships remained unchanged in innervated conditions. All the studies to date that show advances in SkM maturity with innervation have used non-iPSC derived cell types, which may present a phenotype with already more advanced maturity better primed to mature in response to innervation. In line with existing models, our data show spontaneous baseline activity of MN-SkM EMTs in the absence of any stimulation (Fig. 3F), although this phenomenon is not consistent and in many cases was observed to be absent from MN-SkM EMTs that showed robust blue light responses. We conclude that spontaneous activity, should not be used to rule out innervation in 3D models, despite being an excellent positive marker of innervation, as many innervated tissues do not display this phenotype. Finally, MN-SkM EMTs show robust and quantifiable response to blue light activation, with SkM-only controls showing no activation, providing confidence that incident blue light acts through MNs expressing ChR2 and not another off target non-specific activation mechanism (Fig. 3G-J). The twitch force of blue-light-evoked response, averaging at 11.0 ± 6.6 % of twitch force elicited from broad field electrical stimulation, suggests that innervation levels in these tissues could be further improved. Histological data further support this point as many muscle fibers observed were found to support AChR clusters with no colocalized SV2 staining. Potential improvements may include adjusting seeding times or densities, or applying specified exercise protocols to encourage innervation, as *in vivo* data clearly supports activity being key in stable innervation (Soendenbroe et al., 2021, Sirago et al., 2023, Soendenbroe, 2022).

As a large, accessible synapse, the NMJ has been extensively imaged in the literature and is known to possess key morphological characteristics that define a mature healthy synapse (Davis et al., 2022). While to some degree these markers differ between species, with size and morphology being identified as key distinguishing features (Boehm et al., 2020), extensive studies have identified commonalities in NMJ maturation, including sealed synaptic clefts and postsynaptic membrane folding (Gautam et al., 1995, Mech et al., 2020, Inoue et al., 2009, Witzemann, 2006). However, achieving these levels of maturation *in vitro* has yet to be realized. In almost all publications of NMJ modeling, simple colocalization of pre- and postsynaptic markers is used to indicate NMJ formation, and in some studies, AChR clustering alone is considered indicative of NMJs, even though AChR clustering, or “pre-patterning”, is an established process that occurs during development prior to neuronal innervation (Yang et al., 2001). Often, colocalization is interpreted as NMJ formation even when the axons are simply running over an AChR cluster. When examining the synapses produced within the MN-SkM EMTs developed here, we identified striated myotubes with characteristic punctate AChR staining throughout the tissues (Fig. 4A-B). We also observed multiple instances of colocalization of the presynaptic protein SV2 with AChR clusters, including instances where motor neurons appear to terminate on the AChR cluster (Fig. 4B-C). However, in mature NMJs, colocalization of AChRs and SV2 is observed to be absolute with complete symmetry pre- and postsynaptically. In our MN-SkM EMTs we see strong colocalization but not complete symmetry, likely indicative of immature *in vitro* synapses with incompletely sealed synaptic clefts (Gautam et al., 1995, Mech et al., 2020, Inoue et al., 2009, Witzemann, 2006, Vemula et al., 2024, Mejia Maza et al., 2021). This imaging gives insight into the level of maturity of the synapses created, suggesting initial structural assembly of contractile machinery, indicated by myotube striations and immature synapse formation characterized by asymmetric colocalization of pre- and postsynaptic markers. We would expect interventions that increase levels of innervation, or inclusion of additional cell types such as terminal Schwann cells, to progress synaptic maturity and may be important in applications that require sealed synaptic clefts or fully mature morphology.

In the native NMJ, ACh is released from the MN in synaptic vesicles and activates the postsynaptic nAChR. To ensure that blue light evoked function in MN-SkM EMTs was driven through this mechanism, we explored the effects of both pre- and postsynaptic neurotoxins, which selectively target machinery involved in release of vesicles or binding to nAChRs. The presynaptic toxin BoT was chosen as it encompasses a range of important proteins in the presynaptic machinery for its action and therefore biologically relevant data confirms assembly of a range of important presynaptic proteins. For postsynaptic toxins, we selected AChM and Tubocurarine, both fast acting toxins that should display wash in/washout effects and demonstrate a key advantage of models such as ours, allowing facile design of washout experiments. BoT/A showed a dose- and time-dependent reduction in function to complete loss of function following intoxication (Fig. 5A-C). Time-dependent intoxication of BoT/B was also shown. BoT/B acts through a different family of proteins in the presynaptic membrane to BoT/A (Rossetto et al., 2014, Elliott et al., 2019, Strotmeier et al., 2012), further validating the assembly of presynaptic machinery with this pan-serotype sensitivity. Long timecourse functional measurements are a strength of this non-invasive, magnetic sensing technology and is a key phenotype of BoT intoxication, with patients rarely presenting with botulism before 24–48 hrs following exposure (Lonati et al., 2020). Our system accurately modeled the expected functional deficits from BoT intoxication, and furthermore functional restoration began 2 weeks after BoT/A removal, allowing recovery of function to be modelled in the future (Fig. 5D). For this study BoT/A toxin complex subtype 1 was selected as this toxin subtype is most commonly found in therapeutic products. However, it is well recognized that subtypes of BoT/A demonstrate different kinetics of functional blockade, with BoT/A-3 showing much more rapid loss of blockade compared to other subtypes (Pellett et al., 2018, Pellett et al., 2015). Additionally, BoT/E has a more rapid onset of blockade and shorter half-life of action and for this reason is being considered as a therapeutic alternative to BoT/A, as it would have a more favorable safety profile (Martin et al., 2024, Pons et al., 2019). The observation that BoT/A-1 shows persistence within this model suggests that these differences in blockade half-lives could be potentially modelled by this system and would potentially be a powerful future use of the model. Contrary to the long-lasting effects of BoTs, AChM and tubocurarine showed rapid action; ablating NMJ function in < 2 mins of addition. Removal of both toxins (AChM and tubocurarine) showed washout of functional blockade within 20 mins (Fig. 6A-B), showing no lasting effect on NMJ function in line with *in vivo* data (Bowman, 2006, Szalados et al., n.d.). AChM acts as an irreversible inhibitor of the metabotropic AChR, but less potent is this irreversible action on the nAChR at the NMJ supporting the washout of AChM presented here (Hudgins and Stubbins, 1972), which showed depressed function following washout but at a statistically insignificant level. Taken together, the pan-serotype activity of BoTs and the inhibition of NMJ function at the post synaptic membrane confirms that the light-evoked MN-SkM EMT contraction is driven by a biologically relevant mechanism and not a non-specific, cell–cell interaction.

Finally, we observed a clear and uniform dose response to BoT/A at 24 hrs post intoxication (Fig. 5A). The existing lot release procedure for potency testing of BoTs requires the use of mice, in the mouse lethality bioassay (MLB). The MLB poses a series of challenges, inter-site variability can exceed 50 % (Pellett et al., 2019, Sesardic et al., 2003), making unit comparison between toxins impossible, and different serotypes relate differently to human potency; for example, BoT/B is 40x more potent in mice than humans (Strotmeier et al., 2012). Reproducibility of this assay was excellent, the difference of EC_50_ values between 2 different iPSC differentiations of both cell types and users was less than 2 % (Fig. S4B), making an assay like this attractive for potency screening applications. Cell based alternatives for the MLB in 2D formats do exist (Behrensdorf-Nicol et al., 2020, Yang et al., 2024, Fonfria et al., 2023, Hong et al., 2021, Fernández-Salas et al., 2012), although these are focused on a single serotype (often BoT/A) and likely lack the complete biological functional complexity of the NMJ, which may be important to fully capture potency. Validation of cell-based assays such as the one presented must be extensive and robust, the demonstration of key performance criteria and the comparison of these criteria to other existing assays (both *in vitro* and *in vivo*) is fundamentally important before use in regulatory contexts. Initial qualification of this assay would assess response linearity to toxin batch dilutions, multi-user comparisons of performance, multi-site comparisons of performance, specificity with neutralizing antibodies and potency comparisons to MLB results. These tests would utilize purified toxin, however for applications beyond drug product potency, such as diagnostic testing, different sample matrices would also need to be considered, in addition to sample stability and storage considerations. This regulatory framework, while demanding and requiring extensive time and resource commitment, is what ensures potency workflows are effective and fit for purpose in clinical contexts.

## 5 Conclusions

In summary the presented work demonstrates a method by which functional engineered NMJs can be created in a turnkey, reliable, and scalable manner from iPSC MNs and myoblasts. Through the expression of ChR2 in the MNs only, contractile function can be evoked using blue light and compared with total evoked function for estimation of the proportional innervation of engineered tissues. This model is an advancement on previously presented work in its simple consumable and tissue engineering manufacturing processes, allowing tissue generation at scales relevant to preclinical research. This approach additionally benefits from its parallel measurement capabilities, providing a path to collecting large amounts of data without significant challenges or complications. Unlike the majority of existing models, the use of evoked function of our model allows quantifiable metrics to be readily extracted. The dose-dependent sensitivity of the NMJs within these engineered tissues to BoT/A suggests that the platform has utility in potency testing of biologically derived toxins and may serve as a broader proof-of-concept for the utility of 3D models for potency testing. Finally, the broad-spectrum validity of toxin responses and the histological identification of NMJ synapses suggests this system may have future utility in preclinical disease modeling. Diseases that do not derive from genetic defects could be modeled readily in this system, while genetic diseases would require additional work to introduce blue light sensitivity to disease iPSC lines or optimization of viral delivery systems to existing disease lines. To conclude, we present this work with the hope that this turnkey solution to a complex tissue engineering problem can be used to advance preclinical testing in a meaningful way and replace animal models at key moments throughout the drug development process.

**Funding sources**

The work presented within was supported by the National Center for Advancing Translational Sciences of the National Institutes of Health under award number U44TR004795, and by a Weill Neurohub Investigator award to A.S.T.S.

M.C.M. was also supported by the WA state-funded ISCRM Fellows Program (IFP).

## CRediT authorship contribution statement

**Jacob W. Fleming:** Conceptualization, Formal analysis, Investigation, Supervision, Writing – original draft, Writing – review & editing. **Molly C. McCloskey:** Conceptualization, Investigation, Writing – original draft, Writing – review & editing. **Kevin Gray:** Conceptualization, Investigation, Writing – review & editing. **David R. Nash:** Conceptualization, Investigation, Writing – review & editing. **Vincent Leung:** Conceptualization, Investigation, Writing – review & editing. **Christos Michas:** Conceptualization, Visualization, Writing – review & editing. **Shawn M. Luttrell:** Investigation, Writing – review & editing. **Christopher Cavanaugh:** Investigation, Writing – review & editing. **Julie Mathieu:** Investigation, Writing – review & editing. **Shawn Mcquire:** Conceptualization, Project administration, Supervision, Writing – review & editing. **Mark Bothwell:** Project administration, Funding acquisition, Writing – review & editing. **David L. Mack:** Conceptualization, Project administration, Writing – review & editing. **Nicholas A. Geisse:** Conceptualization, Funding acquisition, Project administration, Supervision, Writing – review & editing. **Alec S.T. Smith:** Conceptualization, Formal analysis, Investigation, Funding acquisition, Project administration, Supervision, Writing – review & editing.

## Declaration of competing interest

The author(s) declare the following potential conflicts of interest with respect to the research, authorship, and/or publication of this article: A.S.T.S., and D.L.M. are scientific advisors for Curi Bio, Inc.; the company that commercializes the Mantarray device used in this study. J.W.F., K.G., D.R.N., V.L., C.M., S.M.L., S.M., N.A.G. are employees and equity holders of Curi Bio. All other authors state that they have no conflict of interest (financial or otherwise) associated with this manuscript.

## Data Availability

Data will be made available on request.
