# Supplementary material for: An automated platform for simultaneous, longitudinal analysis of engineered neuromuscular tissues for applications in neurotoxin potency testing
Source: Curr Res Toxicol. 2025 Jan 26;8:100218. doi: 10.1016/j.crtox.2025.100218 (PMC11815696; doi:10.1016/j.crtox.2025.100218)
Supplement: Supplementary Data 1 [file mmc1.docx]

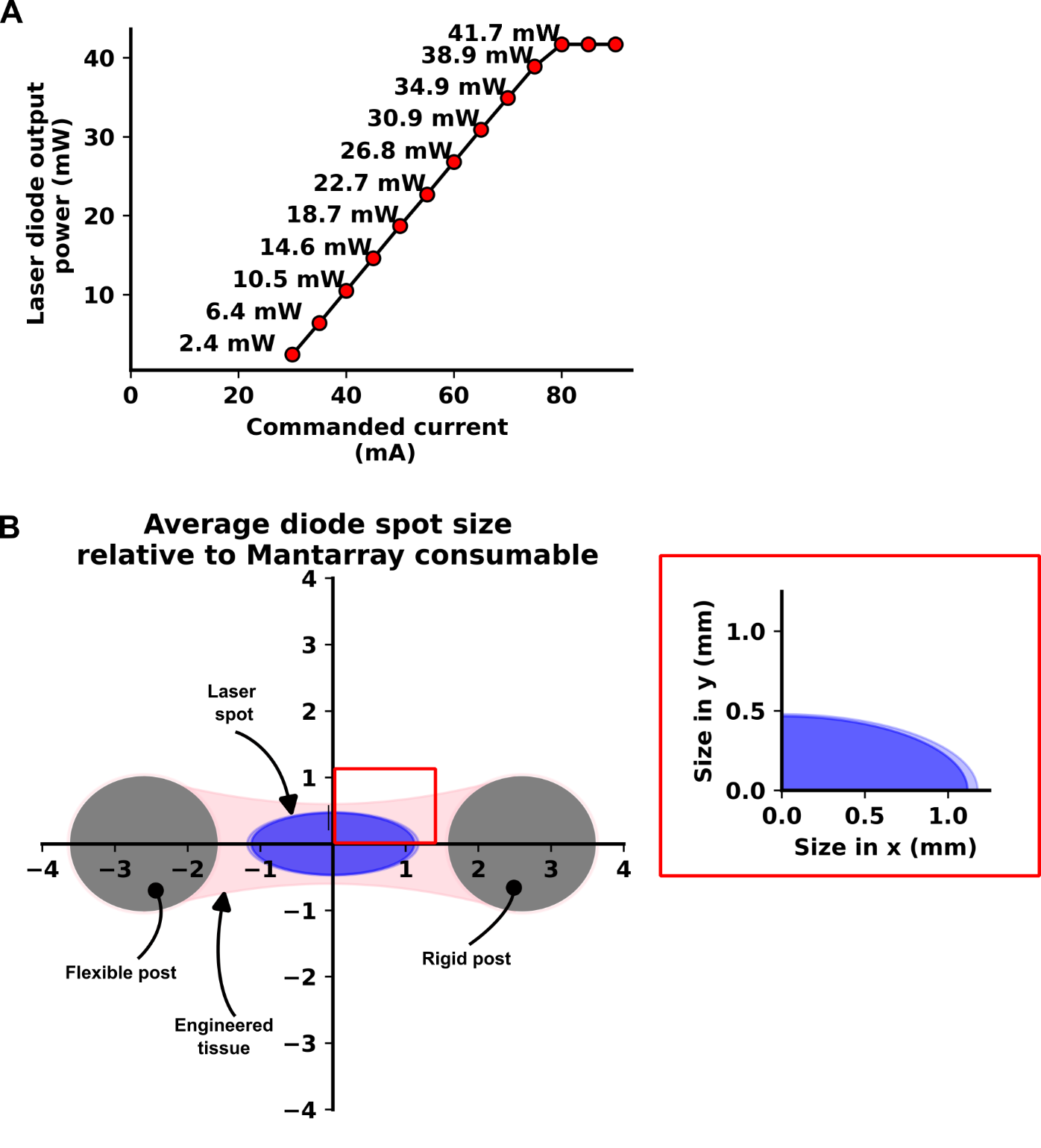


**Figure S1: Characterization of laser diode spot size and power. (A)** Relationship between commanded input current from Mantarray device and laser diode output power. **(B)** Diagrammatic representation of spot size in relation to EMT. Axis scale mm. Red box inset displays mean spot size + S.D. for 24 laser diodes as paler blue ring


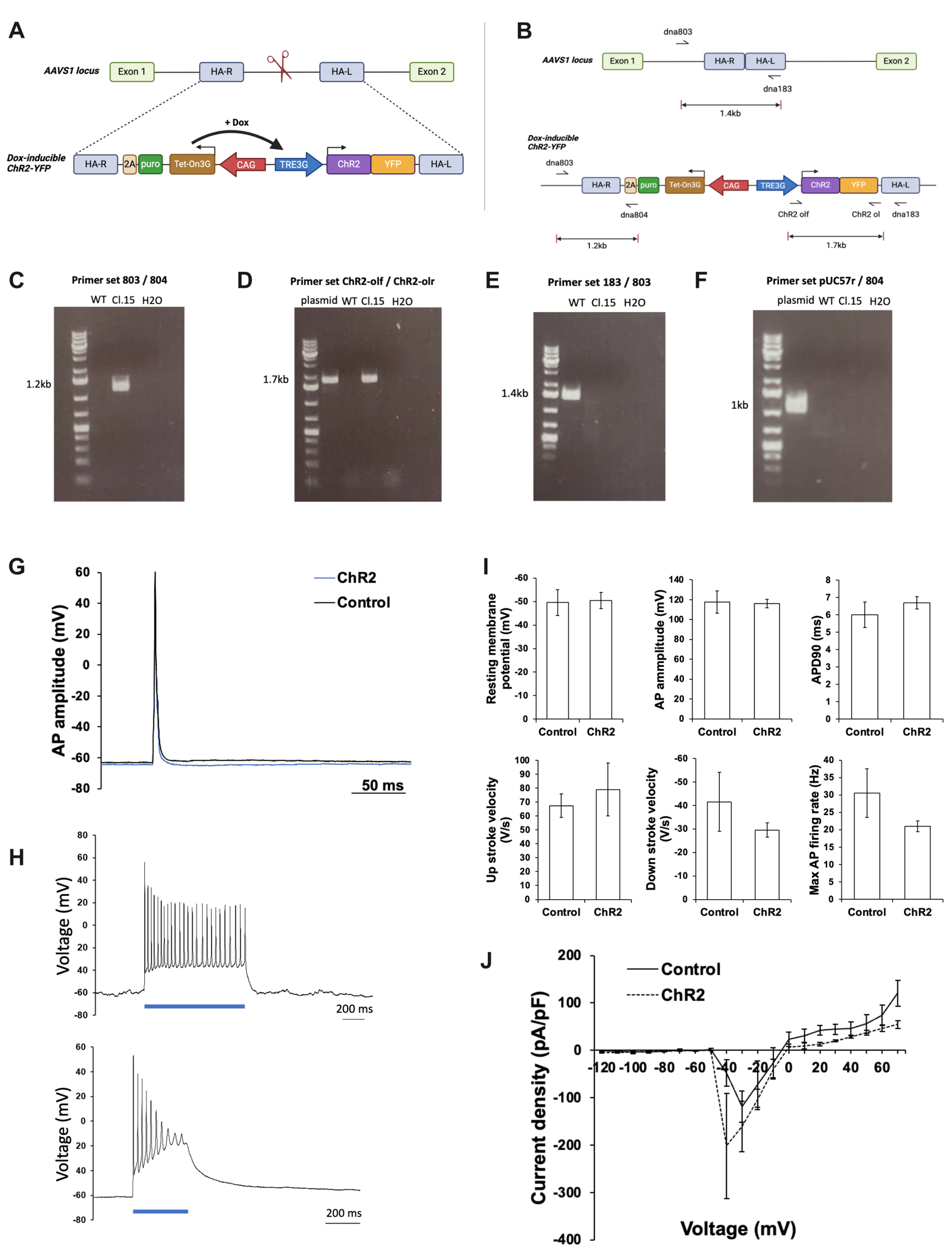


**Figure S2: iPSCs can be transduced with inducible ChR2 transgenes and differentiated into functional and blue light activatable motor neurons (A)** Inducible channelrhodopsin 2 (ChR2)-YFP construct for introduction in AAVS1 safe harbor site. **(B)** Primer sets for PCR to confirm proper transduction of AAVS1 locus and doxocycline (Dox) inducible ChR2-YFP. **(C-F)** PCR evaluation of transduction on WTC11 iPSCs, Clone 15 to confirm (C) insertion of the construct in AAVS1 locus (803 and 804 primer set, expected amplicon size = 1.2kb), (D) presence of the construct (ChR-olf and ChR-olr primer set, expected amplicon size = 1.7kb), (E) bi-allelic vs mono-allelic insertion (183 and 803 primer set, expected amplicon size = 1.4kb for mono-allelic, no band for bi-allelic; Clone 15 is bi-allelic), and (F) random insertion of the plasmid (pUC57r and 804 primer set was used: expected amplicon size = 1.7kb; Clone 15 does not have random insertion). **Abbreviations**; **WT** – Wildtype, WTC11 iPSCs, **Cl. 15** – Clone 15 WTC11 iPSCs. **H2O** – negative control. **(G)** Representative whole cell patch clamp traces of wildtype control (Control) and Cl. 15 gene edited (ChR2) motor neuron action potential (AP) wave forms in response to electrical stimulation. The waveform does not change with insertion of ChR2. **(H)** Example whole cell patch clamp traces of Cl. 15 ChR2 motor neuron action potential firing trains in response to prolonged blue light stimulation. While data indicates cells are capable of firing action potential trains, the cells so somewhat varying levels of maturity, as expected for motor neurons in culture. **(I)** Quantification of current patch clamp metrics demonstrate no major changes in action potential properties with gene editing to introduce ChR2 compared to WT controls. **Abbreviations**; **ChR2** – Channelrhodopsin2, **AP** – action potential, **APD90** – action potential duration at 90% repolarization. **(J)** Whole cell voltage clamp recording of wildtype control (Control) and Cl. 15 gene edited (ChR2) motor neurons, demonstrating similar activation of voltage gated sodium channels occurring around -40 mV as expected. For all plots, mean values ± S.D. are shown. n=4 cells (Control), n=6 cells (ChR2).


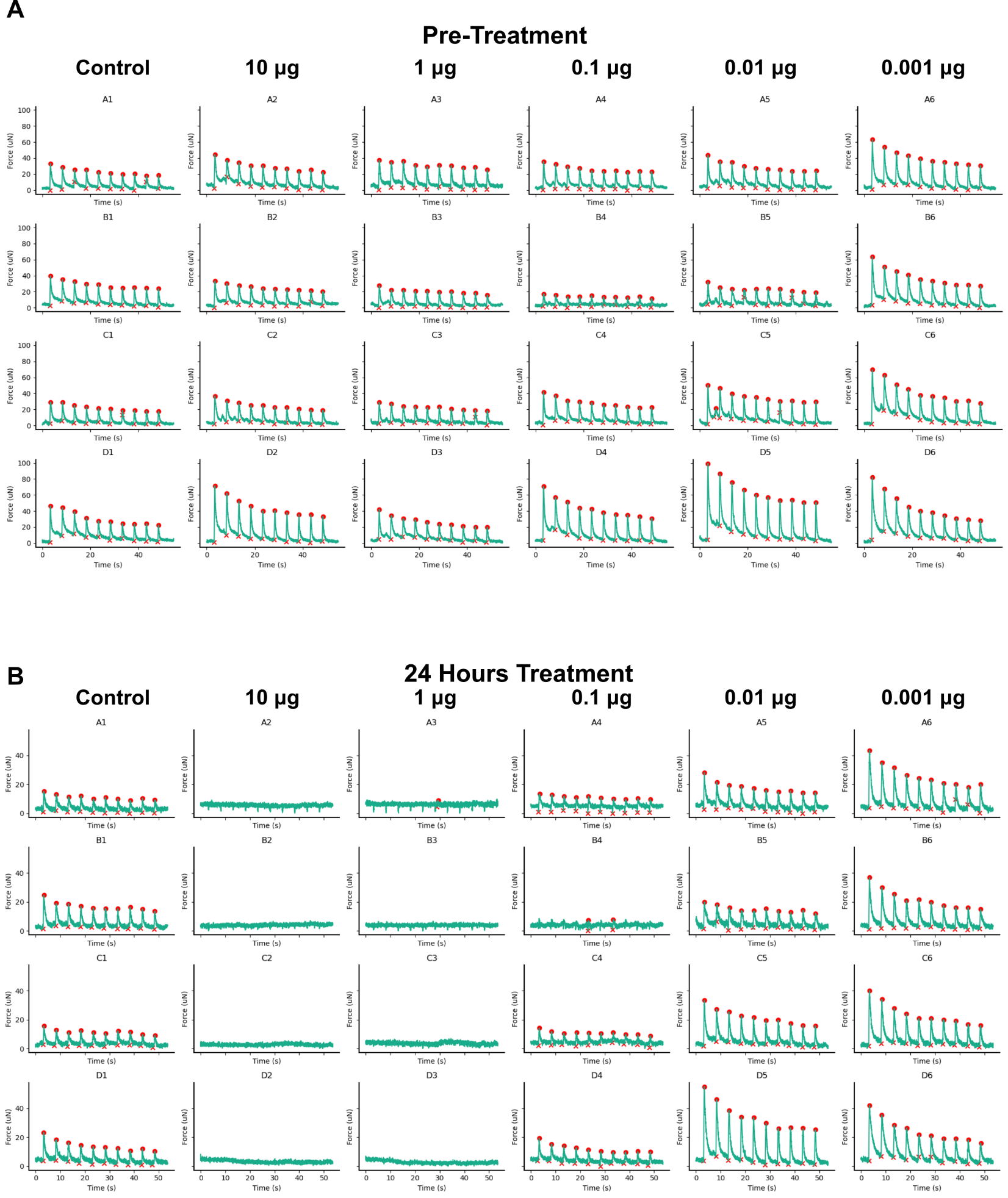


**Figure S3: Reproducibility of BoT response within single plate of MN-SkM EMTs. (A-B)** Individual waveforms from single 24-well plate recording with automatically identified peaks (red circles) and baselines (red crosses) marked. (A) shows recordings from tissues in response to blue light before intoxication, (B) shows the same tissues following intoxication in a dose wise manner decreasing left to right with controls in column 1.


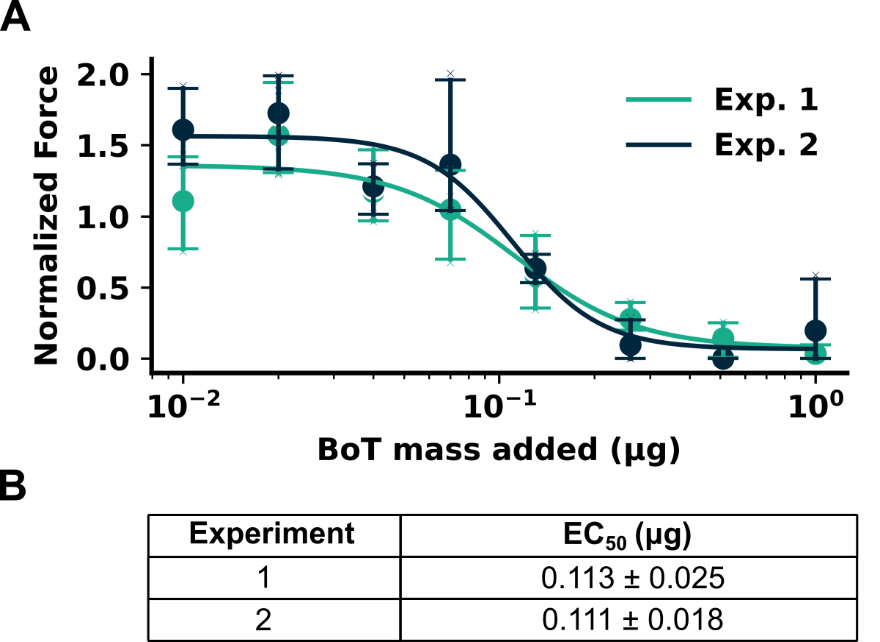


**Figure S4: Reproducibility of BoT response between experiments. (A)** Dose response curves from 2 independent experimental runs using 2 different iPSC differentiations of both cell types and users. **(B)** Quantification of EC_50_ values in an experiment wise fashion. This data is also displayed in an aggregated format in Fig. 5A

**Table S1: Primer sequences for PCR evaluation of AAVS1 transduction.**

| **Primer** | **Sequence** |
| --- | --- |
| pUC57r | ctttatgcttccggctcgtatg |
| Dna183 | CTCAGGTTCTGGGAGAGGGTAG |
| Dna803 | TCGACTTCCCCTCTTCCGATG |
| Dna804 | GAGCCTAGGGCCGGGATTCTC |
| ChR2-olf | ctttccgtaccacttcctaccctcgtaaagtcgacGCCACCATGGACTATGGC |
| ChR2-olr | gtaaccattataagctgcaataaacaagttaacATCATATGTTACTTGTACAGCTCG |

**Table S2: Antibody details for immunohistochemistry**

| **Antibody/Stain** | **Dilution** | **Vendor/Cat No.** |
| --- | --- | --- |
| **Primary antibodies** | | |
| SV2 | 1:300 | Invitrogen / PA5-52476 |
| a-actinin | 1:400 | Invitrogen / MA1-22863 |
| ISL-1 | 1:200 | DHSB / 39.3F7 |
| Tuj-1 | 1:200 | Sigma-Aldrich / T2200 |
| ChAT | 1:100 | Millipore / AB144P |
| MAP2 | 1:1000 | Millipore / AB5622 |
| **Secondary antibodies** | | |
| Goat anti-mouse IgG AF594 | 1:200 | Invitrogen / A-11032 |
| Goat anti-rabbit IgG AF488 | 1:200 | Invitrogen / A-11008 |
| Goat anti-rabbit IgG AF568 | 1:200 | Invitrogen / A-11011 |
| **Molecular stain** | | |
| a-BTX - AF647 | 1:500 | Invitrogen / B35450 |
| DAPI | 1:5000 | Sigma-Aldrich / D9542 |

**Supplemental methods**

**Maintenance of iPSC lines**

Urine-derived iPSC lines generated internally, at the University of Washington, were used for the generation of Curi Bio’s commercial myoblasts. WTC-11 iPSCs [52,53] were used for the generation of TET-ChR2-YFP iPSC line. Both lines were banked in mFreSR medium (Stem Cell Technologies, Vancouver, Canada, 05855) and stored under cryogenic conditions in a vapor phase liquid nitrogen dewar. On the day of plating, vials of cells were removed from liquid nitrogen storage, thawed, pelleted at 300*g* for 3 min, and resuspended in mTeSR medium (Stem Cell Technologies, 100-0276) supplemented with 10 µM Y-27632 (Thermo Fisher Scientific, BDB562822), a specific inhibitor of Rho kinase (ROCK) activity. Cells were then plated on surfaces that had previously been coated overnight with Matrigel (Thermo Fisher Scientific, CB-40234C) diluted 1:60 in DMEM/F12 medium (Thermo Fisher Scientific, 11-320-033). Y-27632 was removed from the culture medium the first day after plating and cells were then fed daily with fresh mTeSR. Cells were incubated at 37°C and 5% CO_2_ until iPSC colonies filled the field of view when visualized using an Eclipse TS100 microscope (Nikon, Tokyo, Japan) fitted with a 10X lens. At this point, cells were lifted off the culture surface using TrypLE Select (Thermo Fisher Scientific, 12563011), collected, spun down, and resuspended in fresh mTeSR with mild trituration to gently break up cell clusters before being split across the desired number of Matrigel-coated plates. During continued culture, any iPSC colonies displaying irregular boundaries, significant space between cells or low nuclear to cytoplasmic ratios were carefully marked and removed from culture using a fire-polished, sterile glass pipette.

**Generation of TET-ChR2-YFP blue light sensitive line**

Working with the University of Washington’s Institute for Stem Cell and Regenerative Medicine Ellison Stem Cell Core, the AAVS1-TRE3-ChR2-YFP construct was generated by cloning ChR2-YFP (PCR from Addgene plasmid #20942) into the pAAVS1-TRE3-GFP construct (Addgene plasmid #52343) using Gibson cloning. One million WTC11 iPSCs [52,53] were electroporated with the resulting AAVS1-TRE3-ChR2-YFP construct (4 µg) and guide RNAs targeting AAVS1 (0.3 µM, Synthego) and Cas9 (1.5 µM, Sigma) as an RNP complex using Amaxa Human Stem Cell Nucleofector (Lonza, kit 2) in the presence of ROCK inhibitor. Two days following the nucleofection, the cells were selected with 0.5 µg/mL puromycin for 3 days. Individual colonies were hand-picked and plated into 96 well plates. Genomic DNA was extracted using Quick Extract DNA extraction solution (Epicentre, QE09050) and clones were screened by PCR using Phusion High Fidelity PCR master mix (Thermo Fisher Scientific, F630). Fig. S2C shows PCR gel results for the selected clone used throughout these studies.

**Electrophysiological characterization of motor neuron activity**

Whole-cell patch clamp recordings were collected from human iPSC-derived motor neurons at day 35-40 post-induction. During recordings, cells were mounted on the 37°C heated stage of an inverted DIC microscope (Nikon) connected to an EPC10 patch clamp amplifier and computer running Patchmaster software (HEKA Eletronik, Lambrecht, Germany). Cells were bathed in a Tyrode’s solution containing: NaCl (140 mM), KCl (5.4 mM), CaCl_2_ (1.8 mM), MgCl_2_ (1 mM), glucose (10 mM), and HEPES (10 mM) (all from Sigma-Aldrich). The intracellular recording solution (L-aspartic acid (120 mM), KCl (20 mM), NaCl (5 mM), MgCl_2_ (1 mM), Mg^2+^-ATP (3 mM), EGTA (5 mM) and HEPES (10 mM); (all from Sigma-Aldrich) was loaded into borosilicate glass patch pipettes (World Precision Instruments, Sarasota, FL, USA). For all recordings, patch pipettes with a resistance in the range of 2-6 MΩ were used and offset potentials were nulled before formation of a GΩ seal. Following the formation of a GΩ seal, suction was applied to disrupt the membrane beneath the pipette, establishing electrical and molecular access to the intracellular space. Membrane potentials were corrected by subtraction of the tip potential, calculated using the HEKA software. Fast and slow capacitance was compensated for prior to each recording.

Both single action potentials and repetitive firing behavior were recorded in current-clamp mode. Five millisecond depolarizing pulses of sufficient intensity (1-2 nA) were applied in order to generate single action potentials for depolarizing waveform characterization analysis. Depolarization-evoked repetitive firing was achieved via application of 500 ms current injections, applied in series, starting at -30 pA and increasing in 10 pA increments. Inward and outward currents were evoked in voltage-clamp mode. This was achieved via a series of 500 ms depolarizing steps from -120 to +30 mV in 10 mV increments. Gap-free recordings of spontaneous activity in patched neurons were performed in current-clamp mode with 0 pA current injection to provide a measure of the resting membrane potential held by the cell without current input. Response to blue-light stimulation was also carried out in this recording mode. Blue-light pulses were generated by a SpectraX light engine (Lumencore, Beaverton, OR, USA) with activation controlled by the Patchmaster software and light delivered to cells through the microscope lens. All recordings and analyses of action potential waveforms and currents were performed using the Patchmaster software suite (HEKA).

Population-level function in motor neuron cultures was assessed in 48-well multielectrode array (MEA) plates using the Maestro Pro MEA system (Axion Biosystems, Atlanta, GA, USA). Human iPSC-derived neurons were passed onto poly-L-ornithine/laminin-coated MEAs at 18 days post-induction and maintained in standard culture conditions until day 25 post-induction. During data acquisition, standard recording settings for spontaneous neuronal spikes were used (Axis software, version 2.5, Axion Biosystems), and cells were maintained at 37°C/ 5% CO_2_ throughout the recording period. The standard settings have 130X gain, and record from 1 to 25,000 Hz, with a low-pass digital filter of 2 kHz for noise reduction. In all experiments, spike detection was set at 5X the standard deviation of the noise. Reported results were calculated by averaging signals from all electrodes in each well, then averaging data from duplicate wells. Blue-light stimulation was achieved using the Lumos hardware (Axion Biosystems) in combination with the Maestro Pro system and controlled using the Axis software. Stimulation parameters were set at 75% maximum light intensity for 50 ms at a stimulation frequency of 1 Hz.
